# Effect of Acupuncture on Body Weight Reduction and Inflammatory Mediators in Egyptian Obese Patients

**DOI:** 10.3889/oamjms.2015.010

**Published:** 2015-01-14

**Authors:** Laila Ahmed Abou Ismail, Alshaymaa Ahmed Ibrahim, Ghada A. Abdel-Latif, Dalia Adel Abd El-Haleem, Ghada Helmy, Lylah M. Labib, M. Kadry El-Masry

**Affiliations:** 1 *National Research Center, Clinical and Chemical Pathology, Cairo, Egypt*; 2 *National Research Center, Community Medicine, Cairo, Egypt*; 3 *National Research Center, Complementary Medicine, Cairo, Egypt*

**Keywords:** Acupuncture, Obesity, IL6, TNF, hsCRP

## Abstract

**AIM:**

Aim of this study was to examine the effectiveness of body acupuncture on body weight loss, routine laboratory tests and pro-inflammatory markers.

**METHODOLOGY:**

The study was performed on eighty obese patients. They were divided into three groups according to their body mass index. Subjects received acupuncture for three- six months in combination with a low-calorie diet. They were assessed pre and post acupuncture, by anthropometric measurement, routine laboratory tests and, tumor necrosis factor- alpha (TNF-α), interleukin- 6 (IL-6), and high sensitivity C-reactive protein (hsCRP) levels in serum.

**RESULTS:**

The pre-acupuncture results showed significant difference between the three grades of obesity and the controls regarding TNFα, IL-6 and hsCRP. We found significant reduction in anthropometric measurement of adiposity after acupuncture. In comparing the pre &post acupuncture results of TNF-α, IL-6 and hsCRP showed high significant reduction after acupuncture. There are highly significant decrease in kidney function (creatinine and uric acid) and lipid profile (cholesterol and triglycerides) and fasting blood glucose, but there was no significant difference in urea, SGPT, SGOT, HDL and LDL.

**CONCLUSION:**

Body acupuncture in combination with diet restriction was found to be effective for weight loss and also reduction of the inflammatory reactions. Acupuncture could be used as a synergistic treatment option for obesity control.

## Introduction

Obesity is a chronic non-communicable disease with clinical and public health challenges [1]. Studies on human and animal models indicated that adipocytes secrete many inflammatory cytokines, and hence the excess of fat in obesity leads to a systemic chronic inflammation [2]. It is now admitted that the majority of inflammatory peptides is secreted by adipose tissue-resident macrophages [3].

Tumor necrosis factor-alpha (TNF-α) and interleukin-6 (IL6) are the most important cytokines responsible for the chronic inflammatory process [4]. In adults, their serum concentrations have been reported to correlate positively with measures of adiposity and to associate with the metabolic syndrome, cardiovascular disease, insulin resistance, and diabetes [2].

High sensitivity C-reactive protein (hsCRP) is a very sensitive marker of inflammation, which is synthesized in the liver, and this process is regulated predominately by IL-6 [4]. hsCRP is positively correlated with abdominal fat and closely correlated with increased risk of cardiovascular events [5].

Acupuncture originated in China more than 2,000 years ago, and is one of the oldest medical procedures in the world. Acupuncture involves the insertion of very fine, sterile needles at specific body points or “energy pathways.” The inserted needles act to stimulate the release of endorphins, the body’s natural “feel good” hormones. This can create a calming, relaxing effect, which counteracts the need for excessive eating brought about by increased stress, frustration or anxiety. In this respect, acupuncture can calm those so afflicted and help them lose weight without resorting to drugs [6].

Recent research has examined some of the mechanisms underpinning acupuncture’s anti-inflammatory effects which include mediation by sympathetic and parasympathetic pathways. Other reported anti-inflammatory effects of acupuncture include an antihistamine action and downregulation of proinflammatory cytokines such as TNF-α, IL-1β, IL-6, and IL-10 [7].

There are several studies which have evaluated the effect of acupuncture in the management of obesity [8, 9]. Acupuncture has been used in the treatment of several diseases including obesity and also immune-related diseases, such as allergic disorders, autoimmune diseases, and immunodeficiency [9]. However, most studies have methodological limitations, including small sample size and inadequately controlled study design [9].

To our knowledge, the effect of acupuncture during body weight loss has not been evaluated with respect to inflammatory and immunological markers such as TNF-α, IL-6 and hsCRP in Egyptian population. Thus, the aim of this study was to examine the effectiveness of body acupuncture on body weight loss, routine laboratory tests and pro-inflammatory markers (TNF-α, IL-6 and hsCRP).

## Methods and Subjects

### Study design

A randomized controlled clinical trial was performed on 80 obese patients entered in our project at National Research Centre. They were divided into 3 groups according to their body mass index (BMI). Subjects received acupuncture for 6 months in combination with a low-calorie diet.

In this study, overweight was defined as a body mass index (BMI) of 25 to < 30 and a BMI of ≥30 were defined as obesity. They neither had received any other weight control measures nor had any medical and/or drug history within the last 3 months before their participation in the study. Only classical Chinese body acupuncture and auricular acupuncture were allowed to be used. Those whom had diabetes, hypertension, heart disease, endocrine abnormalities, or pregnancy were excluded.

Participants were informed about the study both verbally and by written information sheets. Volunteers were given time to discuss the study and were encouraged to ask questions.

Controls were required to be placebo, no treatment, pharmacological or non pharmacological interventions (e.g. diet and exercise).

### Anthropometric Measurements

For all patients, body weight (BW), BMI, and body fat mass were measured by body composition analyzer BC-418 (TANITA, Japan) according to a standard protocol. Height and body weight were measured with the subjects dressed in light clothing after an overnight fasting. The body weight of each subject was measured with a standard scale to an accuracy of ± 0.1 kg, and height was measured to an accuracy of ± 0.1 cm.

### Acupuncture Treatment in Cases

Body points are: Hegu (LI4), Quchi (LI11), Liangmen (ST21), Tianshu (ST25), Zusanli (ST36), Fenglong (ST40), Neiting(ST44), Sanyinjiao(SP6), Daheng (SP15), Neiguan (PC6), Taichong (LR3), Guanyuan (CV4) and Zhongwan (CV12).

Acupuncture sessions was done twice weekly, each session was 30 minutes.

Auricular acupuncture: ear points are: Shenmen, Mouth, Stomach, Sanjiao, Liver, Spleen, Endocrine and Hunger points [10].

They are weekly applied to each ear alternatively.

### Collection of Blood Samples

About 5 ml of venous blood samples were taken from each patient for analysis after a 12-hour fasting, 2 times during the study (at the beginning and 6 months later). Blood samples were collected into vacutainer tubes and centrifuged at 4000 rpm for 10 min. Hemolyzed samples were excluded from analysis. After separation, routine analysis was done and aliquots of serum were frozen at − 80°C for TNF-α, IL-6 and hsCRP analysis.

### Routine Biochemical Analysis and Serum TNF-α, IL-6 and hsCRP analysis

Fasting blood glucose (FBG) was determined immediately on Olympus auto analyzer using hexokinase method. Serum GOT, GPT, urea, creatinine, uric acid (UA), cholesterol, triglycerides (TG) were determined using colorimetric methods on Olympus AU 400 supplied from Olympus Life and Material Science (Europe GmbH, Wendenstraße, Hamburg, Germany).

TNF-α was determined by ELISA [11] kit supplied from Anogen (2355 Derry Road East, Unit 23, Mississauga, Ontario, Canada). Il-6 was determined by ELISA [12] kit supplied from AviBion (Ani Biotech, tiilitie, Finland). Quantitative determination of hsCRP in serum is done by a micro plate immuoezymometric assay [13] supplied from Monobind Inc. (100 North Pointe drive, Lake Forest, USA).

### Statistical analysis

Data was statistically described in terms of mean ± standard deviation (± SD), frequencies (number of cases) and percentages when appropriate. Comparison of numerical variables between the study groups was done using Mann Whitney U test for independent samples when comparing 2 groups and Kruskal Wallis test with Mann Whitney U test as post-hoc multiple 2-group comparisons when comparing more than 2 groups. For comparing pre and post acupunctures, Wilcoxon signed-rank test was used for continuous variables and McNemar test for two related dichotomous variables for detecting changes in responses due to intervention. P value less than 0.05 was considered statistically significant. All statistical calculations were done using computer programs SPSS (Statistical Package for the Social Science; SPSS Inc., Chicago, IL, USA) version 15 for Microsoft Windows.

## Results

Table 1 shows that females represented 93.5% of the total 80 persons. Regarding BMI, obese patients were divided into three groups: obese I (BMI ranged 30 to 35) represented 27.5%, obese 2 (BMI ranged 35 to 40) represented 42.5% and obese 3 whose BMI was > 40 which and represented 30%. Steady pattern of weight gain was reported by about 40% of the studied population.

**Table 1:** General characteristics of the studied group (number (%).

Characters	Total no= 80
**Sex:**	
Male	6 (6.5)
Female	74 (93.5)
**BMI:** (Kg/m^2^)	
30-35 (group1)	22 (27.5)
35-40 (group2)	34 (42,5)
>40 (group3)	24 (30.0)
**Wt. gain pattern:** (%)
Steady	32 (40,0)
Sudden increase	5 (6.3)
Wt. gain after loss	43 (53.7)

There was a significant difference between the three groups of obesity and the controls regarding inflammatory mediators. Routine laboratory results showed that there were no significant differences between three groups of obesity and controls (Table 2).

**Table 2:** Comparison between obesity and control groups regarding routine laboratory tests and levels of inflammatory mediators (TNF-α, IL-6, hsCRP) pre acupuncture (mean ± SD).

Tests	Obese I n=22	Obese 2 n=34	Obese 3 n=24	Controls n=23	P value
Creatinine (mg/dL)	0.7 ± 0.3	0.6 ± 0.4	0.6 ±0 .4	0.9 ±0 .3	0.486

Urea (mg/dL)	33.2 ± 13.1	27.8 ± 13.2	24.5 ± 15.5	35.4 ± 15.6	0.407

SGOT (U/L)	23.6 ± 13.0	22.6 ± 13.4	15.8 ± 12.6	23.7 ± 9.7	0.039

SGPT (U/L)	21.6 ± 11.3	24.7 ± 17.0	15.5 ± 14.7	11.3 ± 6.2	0.072

Fasting blood glucose (mg/dL)	100.9 ± 12.1	97.6 ± 34.7	81.0 ± 44.1	98.0 ± 20.7	0.226

Triglyceride (mg/dL)	140.5 ± 51.5	125.4 ± 74.6	122.2 ± 87.0	135.6 ± 30.9	0.572

Cholesterol (mg/dL)	210.7 ± 50.4	190.8 ± 71.3	176.2 ± 96.6	154.4 ± 1.7	0.885

Uric Acid (mg/dL)	4.9 ± 1.3	5.8 ± 1.5	5.3 ± 1.2	4.4 ± 1.7	0.152

HDL (mg/dL)	45.3 ± 13.5	44.9 ± 11.5	40.1 ± 14.4	50.4±6.9	0.654

LDL (mg/dL)	116.7 ±58.1	118.8 ± 42.7	137.4 ± 34.4	68.6±28.2	0. 590

TNF-α (pg/ml)	16.7 ± 4.7	16.6 ± 4.3	17.5 ± 5.9	11.2 ± 2.2	0.045

IL6 (pg/ml)	16.8± 8.9	15.2 ± 7.1	15.6 ± 8.2	3.8 ± 2.1	0.000

hsCRP (μg/ml)	20.5± 10.9	18.9 ± 11.9	22.5 ± 11.9	5.8 ± 3.9	0.024

Table 3 shows post-acupuncture results. there was no significant difference between three groups of obesity as regards routine lab analysis, except for HDL, FBG and SGPT that showed significant difference (P<0.05).

**Table 3:** Comparison between obesity groups post acupuncture as regards routine lab analysis and inflammatory mediators (TNF-α, IL-6, hsCRP) (mean ± SD).

Tests	Obese I n=22	Obese 2 n=34	Obese 3 n=24	P value
Creatinine (mg/dl)	0.9 ± 0.2	0.8 ± 0.4	0.6 ± 0 .5	0.273
Urea (mg/dl)	29.8 ± 14.6	31.1 ± 17.7	23.9 ± 19.6	0.355
SGOT (U/L)	20.9 ± 11.3	19.7 ± 11.7	14.1 ± 10.7	0.398
SGPT (U/L)	18.9 ± 10.0	19.9 ± 14.5	11.1 ± 10.2	0.032
Fasting blood glucose (mg/dL)	93.0 ± 24.3	81.5 ± 33.6	58.5 ± 39.6	0.029
TG (mg/dL)	117.9 ± 43.8	100.2 ± 62.7	90.9 ± 78.5	0.456
CHOL (mg/dL)	195.0 ± 33.8	165.7 ± 69.6	131.8 ± 89.6	0.208
UA (mg/dL)	4.0 ± 1.1	4.3 ± 2.0	3.2 ± 2.3	0.173
HDL (mg/dL)	72.5 ± 57.1	54.4 ± 33.1	31.4 ± 25.2	0.010
LDL (mg/dL)	110.1 ± 35.0	88.6 ± 59.6	62.9 ± 63.7	0.310
TNF -α (pg/ml)	12.5 ± 2.8	12.6 ± 3.0	13.2 ± 3.8	0.934
IL6 (pg/ml)	14.7 ± 9.3	10.9 ± 7.8	9.0 ± 9.6	0.111
hsCRP (μg/ml)	15.4 ± 7.8	14.3 ± 8.3	18.2 ± 7.4	0.252

Table 4 shows mean level of routine laboratory analysis and inflammatory mediators pre and post acupuncture among obese. There is highly significant difference P<0.01 in kidney function (creatinine & U.A) and lipid profile (cholesterol &TG) and FBG, but no significant difference was found in Urea, SGPT, SGOT, HDL and LDL (P>0.05).

**Table 4:** Comparison between pre and post-acupuncture as regards anthropometric measurements, biochemical characteristics of participants (mean ± SD).

Variable	Pre acupuncture	Post acupuncture	P value
	
Weight (kg)	96.9 ±15.1	91.6 ± 14.6	0.001
BMI (kg/m^2^)	38.6 ± 5.3	36.6 ± 5.0	0.001
Body fat (%)	44.6 ± 52	42.2 ± 5.4	0.001
Fat mass	44.1 ± 11.2	39.6 ± 10.5	0.001
Visceral fat rating	11.8 ± 3.7	10.5 ± 3.7	0.001
Basal metabolic rate	6992.1 ± 926.7	6829.2 ± 836.4	0.001
Creatinin (mg/dL)	0.6 ± 0.4	0.9 ± 1.0	0.001
Urea (mg/dL)	26.6 ± 15.1	29.0 ± 17.5	0.255
GOT (U/L)	19.8 ± 13.6	19.1 ± 11.5	0.451
GPT (U/L)	19.9 ± 14.6	17.9 ± 13.1	0.723
Fasting blood glucose (mg/dL)	91.8 ± 39.3	79.0 ± 35.2	0.001
HDL (mg/dL)	33.8 ± 20.7	37.7 ± 21.4	0.058
LDL (mg/dL)	99.7 ± 64.4	87.3 ± 56.9	0.165
TG (mg/dL)	133.3 ± 80.3	103.9 ± 62.4	0.001
Cholesterol (mg/dL)	186.8 ± 80.5	166.1 ± 70.4	0.001
UA (mg/dL)	4.9 ± 2.4	4.0 ± 2.0	0.001
TNF-α (pg/ml)	16.9 ± 4.7	12.4 ± 3.1	0.001
IL6 (pg/ml)	14.2 ± 8.8	10.4 ± 8.6	0.001
hsCRP (μg/ml)	21.3 ± 9.9	14.9 ± 7.7	0.001

TNF-α, IL6 and hsCRP showed high significant improvement after acupuncture by using non-parametricm (P<0.001).

## Discussion

There are several studies which have evaluated the effect of acupuncture in the management of obesity [9, 14, 15]. Hsu et al., mentioned that waist circumference (WC) is related to the subcutaneous fat tissue of the abdomen, and higher effects of body acupuncture in impolitic activity and enhancing lipid metabolisms could be attributed to the direct effects of body acupuncture in redistribution, lyses of fat tissue and reducing waist circumferences [14]. In current study, different anthropometric measurements in cases showed highly significantly reduction after the acupuncture. A study was done by Hong also observed that acupuncture has the function of selectively reducing excrescent fat in certain parts of the body with extra fat accumulation without further advance after reaching the normal body weight [16]. However, there are some studies that have reported no significant effect of acupuncture in the treatment of obesity, but it should be noted that these studies were performed by auricular acupuncture therapy only [17, 18].

It is believed that acupuncture alters levels of central nervous system by stimulating peripheral nerves at acupoints. Signals are then carried by stimulated nerve resulting in changes in satiety and mood [6]. Acupuncture appears to be able to improve mood by increasing the release of neurotransmitters [19] and suppress appetite by the serotonin and endorphin-induced decreases in stress and depression [20, 21], whereas this effect was not seen by exercise and diet. In addition, it has been shown that application of electroacupuncture at Zusanli (ST-36) and Neiting (ST-44) of the rat caused the increase in the electrical activity of ventral-medical hypothalamus in the obese rat, leading to activation of the satiety center (22).

White adipose tissue (WAT) is a source of some proinflammatory cytokines such as TNF-α and IL-6, which may show both local and systemic effects [23, 24]. Xu et al., also reported that the increased expression of inflammation-specific genes by macrophages in the adipose tissue of obese mice [25].

Many studies showed correlation of TNF- α expression with increased body mass, WC [2, 4, 5]. Previous study on human subjects reported on the elevated levels of TNF-α in gingival crevicular fluid (GCF) of obese individuals. The authors reported on a 0.74αpg increase in GCF TNF-α with an increase of one BMI unit [26]. Moreover, Hotamisligil et al., have shown increased expression of the TNFR2 (TNF receptor 2) receptor in adipose tissue in patients with excessive body weight. They have also demonstrated that the level of the TNFR2 receptor in serum is six times higher in obese people than among their control group [27]. Similarly to TNF-α, serum IL-6 expression correlate with increased body mass, waist circumference, and free fatty acid levels [3, 28] with reduction in circulating IL-6 following weight loss [29]. IL-6 has been implicated as a marker for visceral adiposity because visceral adipose tissue releases more IL-6 than subcutaneous adipose tissue [30].

IL-6 can directly affect lipid metabolism and activate pathways to promote increased energy turnover. IL-6 stimulates lipolysis in humans, increases free fatty acid (FFA) concentrations and whole body fat oxidation adipokines [31]. Notably, IL-6 can decrease the expression and secretion of adiponectin in human adipocytes, as well as other markers of adipocyte differentiation [32]. These findings are in agreement with current study that showed that, in pre acupuncture treatment, there is a significant difference between three grades of obesity and controls regarding TNF-α, IL6. However, in interesting study by Stępie et al., they reported that BMI did not correlate with IL-6 and TNF-α in any investigated subgroups [4]. These findings are in agreement with results obtained by Agraval et al., who also did not observe any significant correlation between IL-6 and TNF-α with BMI in a North Indian healthy general population as well in both sexes [33]. In contrast, Stępie et al., observed a significant inverse correlation between waist to hip ratio (WHR) and TNF-α in the obese male group [4]. Based on the data obtained from this study it is difficult to explain this surprising relationship.

HsCRP is a feature of systemic inflammation and is positively associated with measures of adiposity such as BMI as demonstrated by large two cross-sectional studies [34, 35]. The study by Visser et al., included 8,678 women and demonstrated that obese subjects had CRP 6.21 times higher than those of normal weight [34]. Numerus studies found significant correlation between high level of hsCRP and measures of adiposity [3, 36, 37] and other studies reported reduction in hsCRP levels following weight loss [38, 39].

The effects of the immune system-related acupoints on inflammatory cytokines, such as TNF-α, IL-6 and hsCRP have not been researched sufficiently to date. Although several trials have investigated the influence of some acupoints on the various cytokine levels, there have been no adequately controlled studies comparing the effects of acupoints on the immune system [40]. In this study, we used four of the most common acupoints (Ren12, Li-11, St-36, and Sp-6) that used conventionally in infections and inflammatory diseases. We reported significant reduction of TNF-α and IL-6 after acupuncture. Yim and colleagues reported a decrease in increased serum TNF-α, IL-6, and IFN-γ levels as a result of 3 weekly sessions of electroacupuncture treatment, a total of 5–9 weeks on the St-36 point in collagen-induce arthritic mice. In this study, it was shown that acupuncture could provide a reduction in serum TNF-α level, which was already high in inflammatory arthritis [41]. Likewise, Tian et al., also found that serum TNF-α level increased in rats with ulcerative colitis compared to normal rats. After that, they performed electroacupuncture on the St-36 point once a day for 10 days on these rats [42]. As a result, they reported that there was a decrease in TNF-α level compared to the control rats. On the other hand, Jong and his team were unable to find any changes in IL-4, IL-6, IL-10, soluble interleukin-2 receptor, and IFN-γ levels in 9 healthy individuals in whom they had performed electroacupuncture on the Li-11 point [43]. Karatay et al., mentioned that TNF-α levels that were already expected to be within normal levels in healthy individuals were not affected by the acupuncture treatment in all acupuncture groups in which St-36 and Li-11 were also included [44]. Immediately after a single acupuncture treatment, Petti et al., also reported a significant decrease in IL-10, no significant change in IL-6, and an unexpected significant decrease in IL-2. Since the effects of a single acupuncture treatment are not likely to accurately predict the effects of a substantial course of acupuncture, it is difficult to interpret the results of this study [45].

This study showed significant reduction in hs-CRP level after weight reduction by acupuncture treatment. Many studies have found the same result as ours about the reduction in hsCRP levels following weight loss [4, 46]. Other studies by Hamid et al., found that changes in hs-CRP levels were not different between cases and control, implying this notion that hs-CRP changed independent of the effects of acupuncture [9]. In patients with rheumatologic problems that showed increase hsCRP serum level, some studies reported the inability of acupuncture to reduce hs-CRP levels [47, 48] and some other not [49].

At the end, some potential reasons for these discrepancies among different studies could be due to the differences in age, race, pubertal stage, obesity degree, and fat distribution.

In conclusion, obesity-associated risks factors reduction can be achieved by other modalities but due to lack of adverse events and continued effects after the therapy, acupuncture could be used as a proffered or synergic treatment option for obesity control.
